# Characterization of Pairs of Toxic and Nontoxic Misfolded Protein Oligomers Elucidates the Structural Determinants of Oligomer Toxicity in Protein Misfolding Diseases

**DOI:** 10.1021/acs.accounts.3c00045

**Published:** 2023-04-18

**Authors:** Ryan Limbocker, Nunilo Cremades, Roberta Cascella, Peter M. Tessier, Michele Vendruscolo, Fabrizio Chiti

**Affiliations:** † Department of Chemistry and Life Science, United States Military Academy, West Point, New York 10996, United States; ‡ Institute for Biocomputation and Physics of Complex Systems (BIFI) and Department of Biochemistry and Molecular and Cell Biology, University of Zaragoza, Zaragoza 50009, Spain; § Section of Biochemistry, Department of Experimental and Clinical Biomedical Sciences, University of Florence, Florence 50134, Italy; ∥ Departments of Chemical Engineering, Pharmaceutical Sciences, and Biomedical Engineering, Biointerfaces Institute, University of Michigan, Ann Arbor, Michigan 48109, United States; ⊥ Centre for Misfolding Diseases, Yusuf Hamied Department of Chemistry, University of Cambridge, Cambridge CB2 1EW, United Kingdom

## Abstract

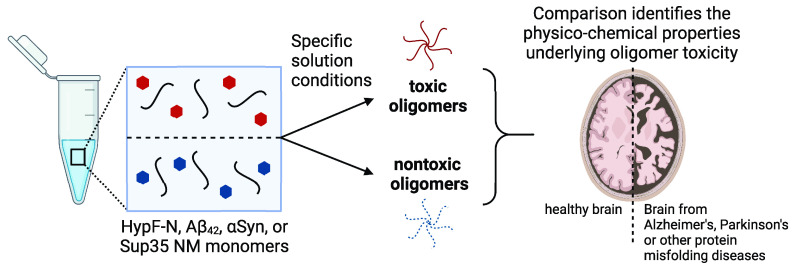

The aberrant misfolding and aggregation of peptides and proteins into amyloid aggregates occurs in over 50 largely incurable protein misfolding diseases. These pathologies include Alzheimer’s and Parkinson’s diseases, which are global medical emergencies owing to their prevalence in increasingly aging populations worldwide. Although the presence of mature amyloid aggregates is a hallmark of such neurodegenerative diseases, misfolded protein oligomers are increasingly recognized as of central importance in the pathogenesis of many of these maladies. These oligomers are small, diffusible species that can form as intermediates in the amyloid fibril formation process or be released by mature fibrils after they are formed. They have been closely associated with the induction of neuronal dysfunction and cell death. It has proven rather challenging to study these oligomeric species because of their short lifetimes, low concentrations, extensive structural heterogeneity, and challenges associated with producing stable, homogeneous, and reproducible populations. Despite these difficulties, investigators have developed protocols to produce kinetically, chemically, or structurally stabilized homogeneous populations of protein misfolded oligomers from several amyloidogenic peptides and proteins at experimentally ameneable concentrations. Furthermore, procedures have been established to produce morphologically similar but structurally distinct oligomers from the same protein sequence that are either toxic or nontoxic to cells. These tools offer unique opportunities to identify and investigate the structural determinants of oligomer toxicity by a close comparative inspection of their structures and the mechanisms of action through which they cause cell dysfunction.

This Account reviews multidisciplinary results, including from our own groups, obtained by combining chemistry, physics, biochemistry, cell biology, and animal models for pairs of toxic and nontoxic oligomers. We describe oligomers comprised of the amyloid-β peptide, which underlie Alzheimer’s disease, and α-synuclein, which are associated with Parkinson’s disease and other related neurodegenerative pathologies, collectively known as synucleinopathies. Furthermore, we also discuss oligomers formed by the 91-residue N-terminal domain of [NiFe]-hydrogenase maturation factor from *E. coli*, which we use as a model non-disease-related protein, and by an amyloid stretch of Sup35 prion protein from yeast. These oligomeric pairs have become highly useful experimental tools for studying the molecular determinants of toxicity characteristic of protein misfolding diseases. Key properties have been identified that differentiate toxic from nontoxic oligomers in their ability to induce cellular dysfunction. These characteristics include solvent-exposed hydrophobic regions, interactions with membranes, insertion into lipid bilayers, and disruption of plasma membrane integrity. By using these properties, it has been possible to rationalize in model systems the responses to pairs of toxic and nontoxic oligomers. Collectively, these studies provide guidance for the development of efficacious therapeutic strategies to target rationally the cytotoxicity of misfolded protein oligomers in neurodegenerative conditions.

## Key References

- Campioni, S.; Mannini, B.; Zampagni, M.; Pensalfini, A.; Parrini, C.; Evangelisti, E.; Relini, A.; Stefani, M.; Dobson, C. M.; Cecchi, C.; Chiti, F.A causative link between the structure of aberrant protein oligomers and their toxicity. Nat. Chem. Biol.2010, 6 ( (2), ), 140–147. DOI: 10.1038/nchembio.283.20081829(1)*Using a pair of toxic (type A) and nontoxic (type B) HypF-N misfolded protein oligomers, hydrophobic flexibility and exposure are found to be key determinants of oligomer cytotoxicity*.
- Cremades, N.; Cohen, S. I. A.; Deas, E.; Abramov, A. Y.; Chen, A. Y.; Orte, A.; Sandal, M.; Clarke, R. W.; Dunne, P.; Aprile, F. A.; Bertoncini, C. W.; Wood, N. W.; Knowles, T. P. J.; Dobson, C. M.; Klenerman, D.Direct observation of the interconversion of normal and toxic forms of α-synuclein. Cell2012, 149 ( (5), ), 1048–1059. DOI: 10.1016/j.cell.2012.03.037.22632969PMC3383996(2)*Initial oligomers (type A) of α-synuclein, a protein playing a key role in Parkinson’s disease, structurally convert to stable, more compact, more structured, proteinase-K-resistant oligomers (type B) that are more toxic*.
- Fusco, G.; Chen, S. W.; Williamson, P. T. F.; Cascella, R.; Perni, M.; Jarvis, J. A.; Cecchi, C.; Vendruscolo, M.; Chiti, F.; Cremades, N.; Ying, L.; Dobson, C. M.; Simone, A. D.Structural basis of membrane disruption and cellular toxicity by α-synuclein oligomers. Science2017, 358 ( (6369), ), 1440–1443. DOI: 10.1126/science.aan6160.29242346(3)The structural properties of stabilized α-synuclein oligomers (nontoxic type A* and toxic type B*) are identified with solution- and solid-state NMR, including a type B* lipophilic N-terminal element that stimulates membrane interactions and a structured core region that penetrates the lipid bilayer.
- Ladiwala, A. R. A.; Litt, J.; Kane, R. S.; Aucoin, D. S.; Smith, S. O.; Ranjan, S.; Davis, J.; Nostrand, W. E. V.; Tessier, P. M.Conformational differences between two amyloid β oligomers of similar size and dissimilar toxicity. J. Biol. Chem.2012, 287 ( (29), ), 24765–24773. 10.1074/jbc.M111.329763.22547072PMC3397903(4)*Toxic prefibrillar oligomers of Aβ*_*42*_*(A+), a protein playing a key role in Alzheimer’s disease, convert into nontoxic oligomers (A−) of similar size, morphology, and lack of secondary structure, where the latter have, however, less solvent exposed hydrophobic moieties and are incapable of disrupting lipid bilayers and causing cell toxicity*.

## 1 Introduction

The process of protein misfolding and aggregation is central to the etiology of a wide range of neurodegenerative diseases, as well as systemic and localized non-neuropathic pathologies, including Alzheimer’s disease (AD), Parkinson’s disease (PD), Huntington’s disease, type II diabetes, light chain amyloidosis (AL), and spongiform encephalopathies.^5^ Characteristic of these diseases is the conversion of normally soluble proteins into insoluble fibrillar aggregates.^5^ Such fibrils bind amyloidophilic dyes, are stabilized by a network of hydrogen bonds organized in a cross-β structure, and can induce autocatalytic pathways that establish a positive feedback loop for further fibril formation.^5^ Almost any polypeptide chain can access the amyloid state,^6^ and proteins tend to be supersaturated in the cell and on the edge of aggregation.^7^ The protein homeostasis system, also referred to as the proteostasis network (PN), prevents the accumulation of protein deposits in tissues, but this system declines during aging.^8^ This can result in the progressive accumulation of fibrillar aggregates in pathology, such as amyloid-β (Aβ) plaques and neurofibrillary tangles of tau in AD, Lewy bodies of α-synuclein (αSyn) in PD, or amyloid deposits of an immunoglobulin light chain in AL.^5^ Alternatively, mutations of amyloidogenic proteins, or other proteins involved in their formation or homeostasis, can promote their aggregation and give rise to early-onset forms of the same diseases, thus anticipating the natural age-related decline of the PN.^5^

**Figure 1:**
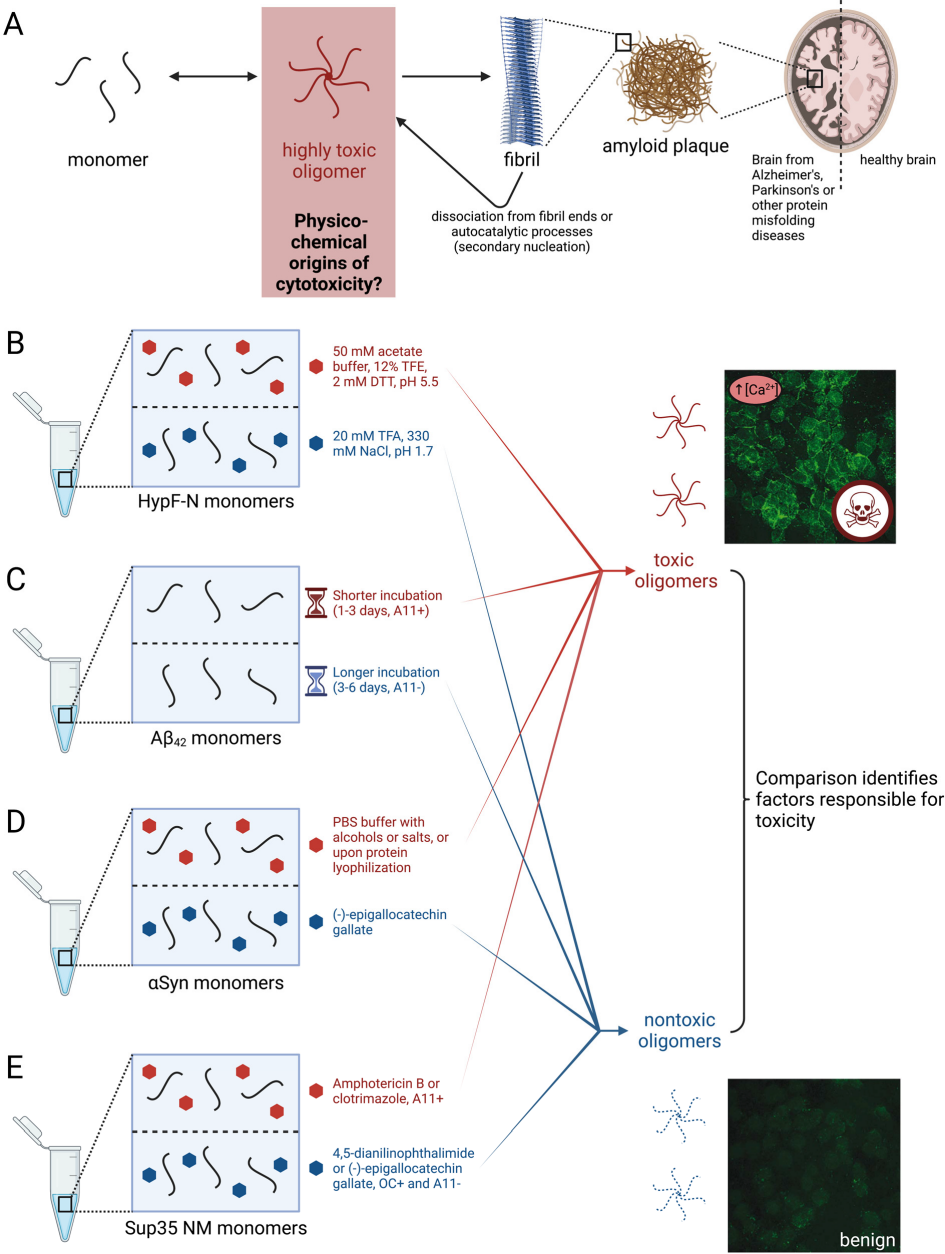
**Illustration of the four pairs of misfolded protein oligomers described in this Account. (A)** Generic schematic for oligomer formation. The solution conditions listed for HypF-N (B), Aβ_42_ (C), αSyn (D), and Sup35 NM (E) lead to the formation of toxic and nontoxic oligomers that facilitate the interrogation of the physico-chemical origins of oligomer cytotoxicity. Ca^2+^ influx images of cells were adapted with permission from ref (1). Copyright 2010 Springer Nature. Created with biorender.com. OC+ and A11+ notes indicate oligomers that react with these specific antibodies. OC– and A11– notes indicate oligomers that do not react with them. Notes with specific compounds (e.g., Amphotericin B) indicate oligomers formed in their presence.

During amyloid fibril formation, transient prefibrillar intermediates known as misfolded protein oligomers are produced in solution (Figure 1A), at first from primary nucleation processes and predominantly from secondary nucleation processes once a critical concentration of fibrils have formed.^9,10^ Oligomers can also accumulate as off-pathway species, representing key pathogenic species in pathology,^11^ or be generated after their detachment from fibril ends.^12,13^ Regardless of their origin, these small, metastable aggregates exist at overall low concentrations and have short lifetimes, as most dissociate back to monomers, convert into higher-order species, such as fibrils, or are cleared by the PN.^5,14^ Nevertheless, oligomers have been detected in AD^15^ and PD brains,^16^ and evidence indicates that elevated oligomer levels are associated with pathology.^17^ The antibody lecanemab, which targets large Aβ oligomers (protofibrils) and slows down cognitive decline in AD,^18^ was recently granted FDA accelerated approval for clinical treatment. A litany of dysfunctional biological responses manifest upon the exposure of cells to toxic oligomers, including membrane perturbation, intracellular Ca^2+^ influx mediated by NMDA and AMPA receptors, mitochondrial dysfunction, reactive oxygen species (ROS) production, lipid peroxidation, an increased caspase-3 response, and aberrant protein–protein interactions, all of which can contribute ultimately to cell death.^19−21^

Extensive work has been devoted over the past two decades toward understanding the structural determinants of oligomer toxicity, including oligomer size, shape, hydrophobicity, secondary structure, structural constraints, compaction, heterogeneity, membrane affinity, and membrane insertion propensity, among many other properties, all hypothesized to be related to their ability to induce cell dysfunction. Critical to addressing these points was the development of pairs of toxic and nontoxic misfolded protein oligomers from the same protein or peptide for a variety of systems, beginning with the 91-residue N-terminal domain of [NiFe]-hydrogenase maturation factor HypF (HypF-N) from *E. coli*,^1^ and then for the 42-residue form of Aβ (Aβ_42_),^4^ αSyn,^2^ and the prion-determining region of yeast Sup35 (NM) (Figure 1B–E).^22^ Following these observations about a decade ago, a series of publications have appeared on pairs of toxic and nontoxic oligomers from these four systems, creating awareness that a comparative structural investigation of each toxic/nontoxic pair could reveal the determinants of oligomer toxicity. Herein, we describe the discovery of these pairs and their structural differences, followed by a discussion on the impact of these molecular tools toward elucidating the physico-chemical properties underpinning oligomer toxicity in a range of pathologies.

## 2 Identification and Initial Characterization of the Oligomer Pairs

### 2.1 HypF-N Oligomers

The first observation of a pair of toxic and nontoxic oligomers formed by the same protein under two different solution conditions came in 2010 from HypF-N,^1^ which is a folded α/β protein domain from *E. coli* with 91 residues and a ferredoxin-like fold.^23^ The two species were generated by incubating native monomeric HypF-N for 4 h at 25 °C at a concentration of 48 μM in either (1) 50 mM acetate buffer, 12% (v/v) trifluoroethanol (TFE), 2 mM DTT, pH 5.5 (toxic Type A condition), or (2) 20 mM trifluoroacetic acid (TFA), 330 mM NaCl, pH 1.7 (nontoxic Type B condition).^1^ After 4 h, the oligomers were sedimented by centrifugation, dried with a gentle nitrogen flow, and resuspended in a more physiological buffer and pH. Importantly, these oligomers were stable for many hours even in the absence of their initial incubation conditions. Both oligomer types weakly bound thioflavin-T (ThT) and possessed similar spherical or discoidal morphologies with diameters of 2–6 nm by atomic force microscopy (AFM), and they therefore shared morphological, structural, and tinctorial properties as oligomers from pathologies like AD and PD.^1^ However, only Type A oligomers were cytotoxic when added to the extracellular medium of human neuroblastoma (SH-SY5Y) or murine endothelial (Hend) cells.^1^ This raised the opportunity of identifying the factors responsible for toxicity through a comparative structural investigation of the two species (see Section 3).

### 2.2 Aβ_42_ Oligomers

Two years later, in 2012, the Aβ_42_ peptide associated with AD was also found to form a pair of toxic and nontoxic oligomers.^4^ The first circumstantial evidence dates back to 2003, when it was reported that Aβ_42_ oligomers could be assembled by incubating 100 μM Aβ_42_ in phenol red-free F12 media at 4–8 °C for 24 h without agitation.^24^ In fact, the investigators observed that the resulting Aβ_42_ oligomers (5 ± 3 nm in diameter) were usually toxic to rat pheochromocytoma PC12 cells, but occasionally they observed Aβ_42_ oligomers with similar size that were no longer toxic, nor were they recognized by oligomer-specific antibodies.^24^ In 2012, this fascinating observation was reproduced using a simpler and more reproducible protocol, which enabled a detailed evaluation of the biochemical properties of the Aβ_42_ oligomers that mediate the dissimilar oligomer toxicities.^4^ The protocol involved incubating 20–25 μM monomeric Aβ_42_ in phosphate buffered saline (PBS), pH 7.4, for up to 6 days without agitation. After 1–3 days, Aβ_42_ formed oligomers (6 ± 1 nm in diameter) that reacted with an oligomer-specific antibody (A11, referred to as A+ oligomers). However, after 3–6 days, the A+ oligomers converted into a second type of oligomer of similar size (6 ± 1 nm in diameter) that no longer reacted with the A11 antibody (referred to as A– oligomers). Importantly, only A+ oligomers were toxic to PC12 cells and primary rat neurons.^4^

### 2.3 αSyn Oligomers

The first reported evidence indicating that αSyn can populate two distinct structural groups with different biological properties came again in 2012 from single-molecule fluorescence experiments.^2^ These experiments directly showed the initial formation, under typical *in vitro* αSyn fibrillation conditions (50–70 μM αSyn, 25 mM Tris, pH 7.4, 0.1 M NaCl, under shaking), of a subgroup of primarily disordered oligomers (named type A oligomers) that slowly converted into a different structural subgroup (designated as type B oligomers), found to be more compact, stable, and protease-resistant with partial β-sheet structure.^2^ These converted oligomers then underwent a progressive increase in β-sheet content upon growth by monomer addition until they reached the fully formed, cross-β structure of the fibrillar species.^25,26^ Only type B oligomers generated high levels of oxidative stress in cultured rat primary neurons compared to other tested αSyn species, including fibrils.^2^ Protocols to obtain structurally homogeneous oligomeric samples of αSyn analogous to both species were later developed, resulting in more than 90% enrichment in each oligomer type. Specifically, treatment of αSyn with the polyphenol (−)-epigallocatechin gallate (EGCG) at 10 molar equivalents in PBS for 48 h at 37 °C promoted the accumulation of unstructured, nontoxic oligomeric forms^3,27^ with spherical-like morphology that behaved similarly as type A oligomers and were coined type A* oligomers.^3^ Alternatively, incubation of αSyn under limited hydration conditions (either PBS with moderate concentrations of alcohols or high concentrations of salts, or upon protein lyophilization in water) promoted αSyn aggregation into structures with an antiparallel intermolecular β-sheet structure and particularly slow conversion to fibrils.^28,29^ These oligomeric forms, particularly those generated upon αSyn lyophilization and resuspension at high protein concentrations (typically 12 mg·mL^–1^), were morphologically and biologically analogous to the type B oligomers, and were thus named type B* oligomers.^28^ Importantly, similar oligomeric species are formed inside neuronal cells,^30^ and analogous levels of stress and dysfunction were observed in PD-related cellular models of αSyn aggregation.^31,32^

### 2.4 Sup35 NM Oligomers

Another important observation was obtained with a fragment of the Sup35 protein from *S. cerevisiae*, which is a translational termination factor and one of several yeast prion proteins.^33^ In its soluble and non-prion state [*psi*^*–*^], the function of Sup35 is to terminate translation. However, in its prion state [*PSI*^+^], Sup35 assembles into amyloid fibrils that are transmitted from mother to daughter cells due to the occasional readthrough of stop codons in auxotrophic markers.^33^ The N-terminal domain (residues 1–123), enriched in uncharged polar residues (Gln, Asn, and Tyr), and the highly charged middle domain (residues 124–253) are referred to as NM and represent the prion-determining region. In 2012, it was reported that two distinct forms of Sup35 NM oligomers could be assembled sequentially by incubating NM (2.5–10 μM) in 5 mM potassium phosphate, 150 mM NaCl, pH 7.4, for 3–5 h.^22^ The first NM oligomers that formed after 30–60 min were recognized by the A11 oligomer-specific antibody, but not the OC antibody that is more specific to late oligomers or fibrils. They were soluble and readily dissociated using a strong surfactant, such as sodium docecyl sulfate (SDS). A second population of NM oligomers formed at 60–75 min, were recognized by the OC antibody, and they were also soluble and dissociated by SDS. Neither of these oligomers were detected at later assembly times (>100 min). To overcome their kinetic instability, small molecules were identified to selectively stabilize each NM oligomer.^22^ Amphotericin B and clotrimazole at a 4-fold molar excess stabilized toxic A11+ oligomers and prevented their conversion into OC+ oligomers. Conversely, 4,5-dianilinophthalimide and EGCG stabilized nontoxic OC+ oligomers that were not recognized by the A11 antibody.

## 3 Insights Obtained from HypF-N Oligomers

Following these first observations on four different proteins, the HypF-N oligomer pair was carefully investigated. Morphologically, both HypF-N oligomer types were found, using AFM, to be spheroidal or discoidal, with diameters of about 2–6 nm.^1^ They possess significant and similar contents of β-sheet structure^34,35^ and display weak ThT binding,^1,35^ although to a slightly higher extent for the toxic type A oligomers suggesting a more compact structure.^35^

Despite these similarities, their molecular characterization revealed important differences. In a first study, 18 mutants of HypF-N were produced with a single cysteine residue at a given position, and then labeled with *N*-(1-pyrene)maleimide so that the fluorophore labeled only the cysteine residue.^1^ The 18 labeled mutants were oligomerized into type A and B oligomers, and their pyrene excimer emission intensities, as well as their values of ratio of intensities of the I and III bands of pyrene (*I*_I_/*I*_III_), were determined to report on the structural order and solvent exposure of the labeled residue within the oligomers, respectively. Nontoxic type B oligomers were stabilized by intermolecular interactions between the three major hydrophobic regions of the sequence, such that a lower fraction of the hydrophobic residues were solvent-exposed on the oligomer surface relative to toxic type A species.^1^ Toxic type A oligomers also bound more strongly the 8-anilinonaphthalene-1-sulfonic acid (ANS) probe, which binds preferentially to clusters of solvent-exposed hydrophobic residues.^1^ Collectively, toxic oligomers demonstrated lower hydrophobic packing correlated with a greater ability to penetrate the cell and induce dysfunction. Hydrophobic exposure was therefore identified as a key determinant of oligomer toxicity.^1^

Subsequently, solution-state and solid-state nuclear magnetic resonance (NMR) spectroscopy showed that toxic oligomers had a highly organized core, an overall greater compactness, extensive hydrogen bonding and greater structural rigidity than nontoxic oligomers.^35^ Toxic oligomers also had a structured N-terminus, unlike toxic ones.^35^ Site-directed fluorescence resonance energy transfer (FRET) experiments, involving two different cysteine residues from different protein molecules labeled with donor (D) and acceptor (A) for a total of ca. 50 FRET pairs per oligomer type, showed higher FRET efficiency values, on average, for toxic species, confirming an overall greater compactness and structural rigidity relative to nontoxic species.^35^ However, FRET efficiency values were, on average, lower in toxic than nontoxic oligomers when D and A were both placed on hydrophobic residues, indicating a lower level of hydrophobic interactions in toxic species. Hence, the higher compactness and rigidity of the toxic misfolded oligomers generates structural constraints that cause the hydrophobic residues to interact less strongly with each other, with a fraction of them becoming exposed to the solvent.^35^ These solvent-exposed hydrophobic residues, which are also present in the nontoxic oligomer form but to a remarkably lower extent, were proposed to mediate the ability of the toxic oligomers to drive membrane interactions, resulting in not only their interaction with, but also the aberrant destabilization of the cell and its homeostatic processes.^1,35^

In fact, when the oligomers were added to the extracellular medium of cultured cells, to the medium of *C. elegans* worms or injected into rat brains, only type A oligomers were toxic.^1,20,36,37^ The binding of oligomers to the bilayer of cell membranes, observed predominantly for toxic species and requiring the presence of the ganglioside GM1,^38^ is thought to represent an important molecular event in the mechanism through which these oligomeric species manifest their toxicity, because a massive influx of calcium ions (Ca^2+^) from the extracellular medium to the cytosol is triggered by this binding, mediated by either NMDA or AMPA receptors acting as calcium channels or nonspecifically through the membrane.^20,38−40^ The Ca^2+^ influx leads in turn to a number of deleterious events, such as ROS formation, lipoperoxidation, mitochondrial dysfunction, further membrane destabilization, and eventually apoptosis^20,40^ and, in the case of primary neurons, hippocampal slices, and whole animals, also affect cholinergic neuronal cells^20^ and lead to colocalization and disruption of post-synaptic densities, impairment of long-term potentiation (LTP), and impairment of rat spatial memory, as assessed using the Morris Water Maze.^36^

Studies with AFM and supported lipid bilayers (SLBs) showed that only toxic type A oligomers bound the bilayer, and binding associated with toxicity occurred within the GM1-enriched gel-phase domains (Lβ or So), as opposed to the liquid-disordered phase domains (Lα or Ld) of the SLBs.^41^ By labeling the oligomers with a fluorophore that changes its fluorescence spectrum when transferring from the bulk solvent to the membrane, the binding affinity of the oligomers for reconstituted liposomes (LUVs) was determined, and the dissociation constant (*K*_D_) value of the oligomers–liposomes complex was found to be 25-fold higher for the toxic species.^42^ Similarly, the collisional quenching of the oligomers with the lipid membrane, quantified by embedding a suitable fluorophore within the membrane bilayer and measuring the Stern–Volmer constant (*K*_SV_), was found to be 20-fold higher for the toxic oligomers.^42^ It was also found that neither oligomer type exhibited structural changes upon interaction with lipid membranes, and toxic oligomers did not feature a preferential binding to any of the lipids contained in LUVs.^42^

## 4 Insights Obtained from Aβ_42_ Oligomers

Comparison of the similarly sized toxic A+ and nontoxic A– Aβ_42_ oligomers also revealed key molecular insights into their properties.^4^ First, the two oligomers possessed large differences in hydrophobicity, as determined using a proteolytic assay and sequence-specific antibodies directed against various portions of the sequence, among other simpler assays. In addition, toxic A+ oligomers were found to be less stable than A– oligomers.^4^ This increased hydrophobicity and instability resulted in an enhanced ability to disrupt synthetic (l-α-phosphocholine) lipid membranes *in vitro*, as measured by an increase in lipid bilayer conductance.

The A+ oligomers unfolded at lower denaturant concentrations than the A– oligomers and markedly lower concentrations than Aβ_42_ fibrils. The lower stability of A+ oligomers resulted from the increased solvent accessibility of its hydrophobic peptide segments.^4^ The relative solvent accessibility of different linear epitopes in Aβ_42_ for each oligomer type was evaluated by incubating them with Proteinase K and periodically depositing the samples on nitrocellulose membranes to quench the reactions. The ability of a panel of Aβ antibodies with linear epitopes to recognize the proteolyzed oligomers as a function of reaction time revealed unique patterns of relative solvent accessibility. The hydrophilic N-terminus of A+ and A– oligomers (Aβ_42_ residues 3–10) was proteolyzed at the same rate as Aβ_42_ monomers and fibrils, suggesting a lack of structure in each case. In contrast, the hydrophobic middle (Aβ_42_ residues 16–21 and 18–22) and C-terminal (Aβ_42_ residues 30–35, 35–39, and 37–42) peptide segments within A+ oligomers were proteolyzed slower than those for Aβ_42_ monomers, but faster than those for A– oligomers and Aβ_42_ fibrils. In addition, A+ oligomers bound to ANS with higher affinity than A– oligomers with a higher blue-shift of its fluorescence (483 nm vs 502 nm, respectively) and were soluble in lithium dodecyl sulfate (LDS), unlike A– oligomers.^4^ These findings suggest that the relatively high solvent accessibility of specific hydrophobic Aβ peptide segments in A+ oligomers mediate their increased hydrophobicity and toxicity.^4^

Two independent studies demonstrated that A+ oligomers bound more strongly to SH-SY5Y cells and mediated cellular dysfunction more effectively than A– oligomers,^38,43^ consistent with the increased hydrophobicity, lower stability, and membrane disruption activity of the A+ oligomers. Furthermore, single molecule tracking experiments demonstrated that A+ and A– oligomers have a rate of similar lateral diffusion on the plasma membrane of living cells (although A– oligomers bind less effectively), but only the toxic A+ oligomers altered the mobility of GM1.^44^ A+ oligomers accumulated in proximity of lipid rafts where both GM1 and membrane NMDA/AMPA receptors are located, inducing early and transient Ca^2+^ influx,^45^ suggesting that both lipid and protein components of the plasma membrane contribute to neuronal dysfunction induced by A+ oligomers.

## 5 Insights Obtained from αSyn Oligomers

Both toxic and nontoxic αSyn oligomers have been reported to exhibit similar spherical morphologies and size distributions (4–5 nm in diameter, ca. 15–40 molecules).^3,12,28^ For secondary structure, nontoxic type A* oligomers were largely disordered, whereas toxic type B* ones contained a β-sheet rich core composed of half of the β-sheet content typically observed in mature αSyn fibrils.^12^ This resulted in a significantly reduced ThT binding, as compared to the fibrillar form, although it showed the typical cross-β X-ray diffraction pattern, with an inter-strand spacing of 4.6 Å and an inter-sheet distance of 8.9 Å.^12^ The weak ThT binding reflects the deficiencies in regularity and compactness of the cross-β structure in the type B* oligomers relative to fibrils, although they showed high chemical stability,^28^ comparable to that of the fibrils. Type B* oligomers also showed a positive correlation between size and β-sheet content, and the presence of a minimum oligomer size, approximately 200 kDa, below which type B* oligomers are no longer stable and rapidly disassemble into monomers.^28^ Importantly, only type B* oligomers strongly bound to ANS, indicating a high degree of solvent-exposed hydrophobicity, and were positive to the conformation-sensitive A11 antibody.^12^

The structural homogeneity of both type A* and type B* oligomeric samples allowed their structural characterization by solution-state and solid-state NMR.^3^ The interaction of EGCG with monomeric and oligomeric αSyn has also been studied extensively by solution-state NMR.^46^ αSyn is disordered in type A* oligomers, and the disordered core included the N-terminal region (residues 1–36) among other sequence regions. In contrast, the β-sheet core of type B* oligomers included the most amyloidogenic region (residues 70–88), while the N-terminal segment of the protein remained disordered.^3^

The interaction of both oligomer types with lipid membranes of cells was probed by a range of NMR techniques.^3^ Toxic type B* oligomers established strong bilayer interactions through its solvent exposed N-terminal segment (residues 1–26); once anchored on the membrane surface, similarly to the mechanism described for the monomeric form, the hydrophobic β-sheet core is inserted in the interior of the bilayer, causing major membrane disruption.^3,12,28,32,47^ Type A* oligomers, in contrast, interacted with the membrane surface in an unspecific manner and without apparent membrane insertion.^3,12^ A follow-up study reported that long or short αSyn fibrils (ca. 500 and 50 nm, respectively) also interact with membranes but are unable to insert their β-sheet cores in the interior of lipid bilayers as type B* oligomers do.^12^

The ability of type B* oligomers to insert into lipid bilayers makes them particularly toxic to cells, including primary neurons,^3,12,31,32,47^ whereas monomers and type A* oligomers are biologically inert under analogous conditions.^3,12^ The strong perturbation of the plasma membrane induced by type B* species resulted in oligomer internalization and concomitant aberrant Ca^2+^ influx into the cell.^12,32^ The impairment of ion homeostasis promotes in turn oxidative stress by increasing ROS production and decreasing endogenous glutathione.^3,12,31^ This activates apoptotic cascades and aberrant mitochondrial functions that ultimately results in primary neuron death.^3,12,31,32^

Similar effects were observed when cells were exposed to short αSyn fibrillar species, although to a lower extent and with significant delay with respect to type B* oligomers.^12^ Fibril-induced toxicity was mostly associated with the release of type B* oligomers from fibril ends, indicating that the fibrillar species can act as a source of harmful soluble oligomers resembling the intermediate conformers formed *de novo* during aggregation, and whose lifespan is therefore greatly expanded. In particular, neurons exposed to culture medium containing preformed fibrils showed A11-positive, type B*-like αSyn oligomers penetrating into the cytosol.^12,48^ The release of globular oligomeric species from the fibrils was supported by images obtained with super-resolution stimulated emission depletion (STED) microscopy in rat primary cortical neurons. Using either fluorescently labeled fibrils or unlabeled fibrils detected with an antibody specific for exogenous human αSyn, the internalized species observed in neurons showed oligomeric, rather than fibrillar, morphology. Hence, αSyn fibrils, upon interaction with the cell membrane, are able to release type B* oligomers, which are readily internalized into the cytosol.^12,48^

Of note, type B* oligomers are generated through an alternative pathway to that typically associated with the formation of amyloid fibrils,^29^ with the former favoring an antiparallel β-sheet structure, in contrast to the parallel β-sheet configuration expected for the latter process or for oligomers released from the canonical parallel β-sheet fibrils. Both types B and B* oligomers have an intermediate secondary structure between the monomeric and the fully formed mature fibrils and similar sizes, affinities for lipid membranes, and toxic mechanisms, with indistinguishable cellular dysfunction effects.^2,3,28,31,32^ One remarkable difference is, however, their ability to elongate, with type B and type B* oligomers elongating rapidly and remarkably slower, respectively.^49^ Thus, while both types of oligomers behave similarly toxicologically, they are likely to show important differences in terms of seeding and spreading new fibrils.

## 6 Insights Obtained from Sup35 NM Oligomers

The ability to detect toxic A11+ oligomers forming earlier in the assembly process and nontoxic OC+ oligomers forming later for Sup35 NM enabled insights into the molecular origins of their unique properties and toxicities.^22^ First, the A11+ oligomers were less compact than the OC+ oligomers using single-molecule fluorescence measurements (Q38C mutant labeled with Alexa-488, Cy3B, or Cy5). The attached dyes exhibited a higher fluorescence anisotropy in toxic A11+ oligomers than in nontoxic OC+ oligomers. Moreover, the Cy3B dye, that is highly susceptible to tyrosine-mediated quenching, also showed higher fluorescence in A11+ oligomers. These experiments revealed that the A11+ oligomers formed first, with relatively little order and compactness (as indicated by high fluorescence anisotropy and low fluorescence quenching, typically associated with these structural traits^50^), and then OC+ oligomers formed next with increased order and compactness (lower anisotropy and higher quenching) and displayed unique fluorescence properties (quenching and anisotropy) relative to fibrils. Unlike A+ and A– oligomers of Aβ_42_, these A11+ and OC– oligomers showed similar solubilities in SDS.

Further insight was obtained with NM oligomers stabilized with small molecular inhibitors.^22^ Toxic A11+ oligomers stabilized by amphotericin B or clotrimazole were 3- to 6-fold more hydrophobic than NM amyloid fibrils, as detected using Nile red fluorescence. In contrast, the nontoxic OC+ oligomers stabilized by 4,5-dianilinophthalimide or EGCG were less hydrophobic than NM fibrils. These results collectively suggest that toxic A11+ NM oligomers are less compact and more hydrophobic than OC+ NM oligomers, and these differences mediate their large differences in toxicity.^22^

## 7 Conclusions and Outlook

**Figure 2:**
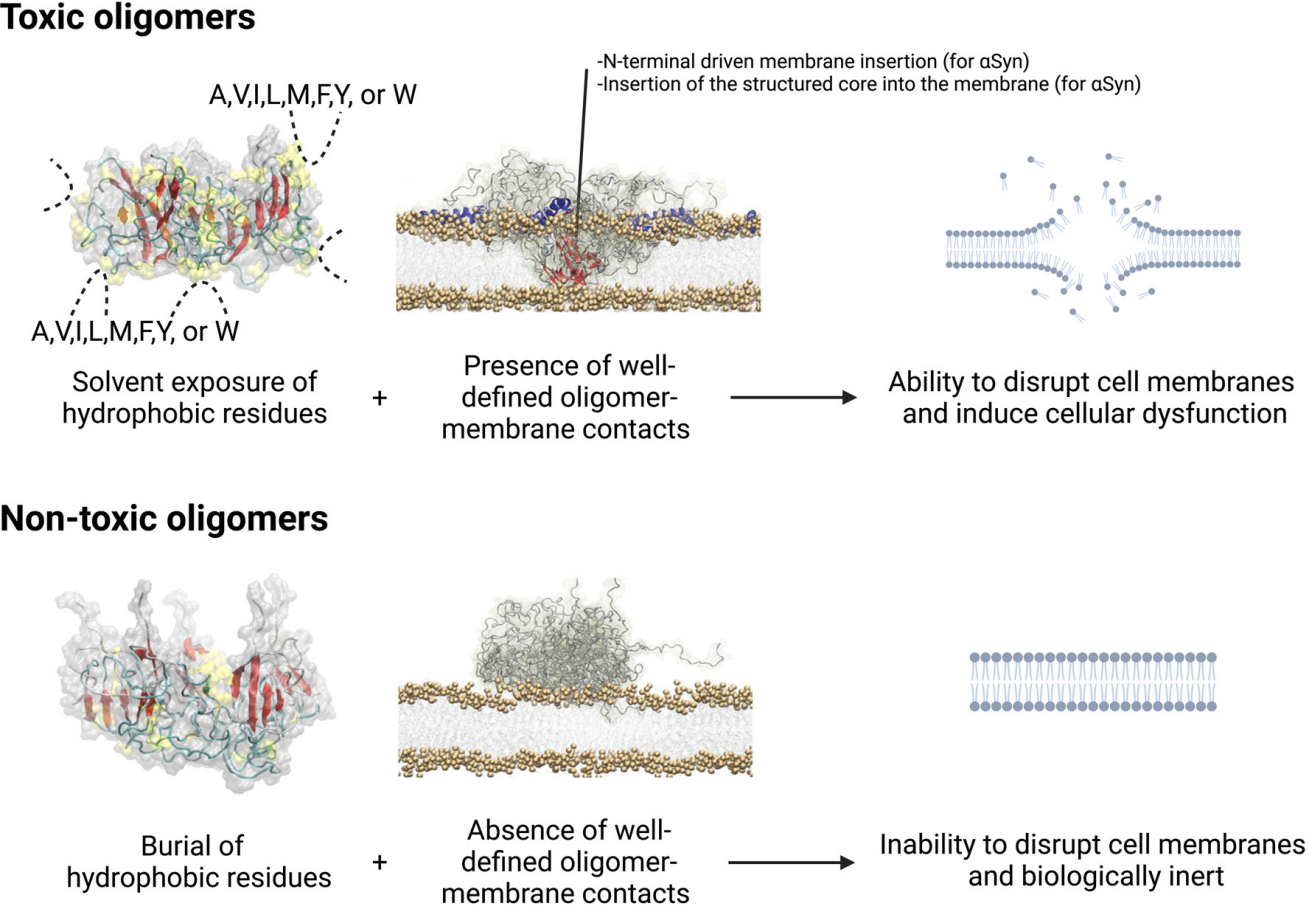
**Physico-chemical factors regulating oligomer toxicity discussed in this Account.** The cell binding mechanism refers to toxic type B* and nontoxic type A* oligomers of αSyn, but is also representative of other oligomer pairs with shared features. Oligomer models (left) are adapted with permission from ref (35). Copyright 2018 Royal Society of Chemistry. Oligomer–membrane models (middle) are adapted with permission from ref (3). Copyright 2017 American Association for the Advancement of Science. Created with biorender.com.

Pairs of stabilized toxic and nontoxic HypF-N, Aβ_42_, αSyn, and Sup35 NM oligomers are enabling the elucidation of the structural properties responsible for oligomer-induced cellular dysfunction in protein misfolding diseases. These properties are summarized in Table 1, whereas the various experimental readouts for determining oligomer toxicity are summarized in Table 2. Collectively, common traits exist for toxic oligomers, including a higher fraction of solvent-exposed hydrophobic residues, a high affinity for biological membranes resulting in an ability to disrupt them, and the capacity to induce cellular dysfunction (Table 1, Figure 2). Reduced stability was observed for the toxic oligomers of Aβ_42_, but not αSyn or Sup35 NM, while a more ordered core was observed for toxic HypF-N and αSyn oligomers, but not other toxic oligomers (Table 1). The β-sheet content does not generally correlate with toxicity, either, because toxic and nontoxic oligomers have often a similar level of this secondary structure type, which is high for HypF-N oligomers, but poor for Aβ_42_ oligomers (Table 1), as observed previously.^34^ Therefore, the presence of solvent-exposed hydrophobic residues appears to be a key shared property of toxic oligomers. Oligomer size has also been found to correlate inversely with oligomer toxicity,^5^ but in most of the oligomer pairs described here the two species have a similar size (Table 1), allowing other determinants to be disclosed.

**Table 1:** Physico-chemical Characteristics of Toxic Oligomers Relative to Nontoxic Oligomers from the Four Protein Systems Described (n.d. = Not Determined)

Studied feature	HypF-N	Aβ_42_	αSyn	Sup35 NM
Exposed hydrophobicity	higher^1^	higher^4^	higher^12^	higher^22^
Compactness	higher^35^	n.d.	higher^12,28^	lower^22^
β-sheet content	similar (high content)^34,35^	similar (poor content)^4^	higher (modest content)^12^	n.d.
Structural level of N-terminus	more structured N-terminus^35^	similarly exposed N-terminus^4^	less structured N-terminus^3^	n.d.
Structural level of the core	more organized core^35^	n.d.	more organized core^3^	less organized core^22^
Stability	n.d.	lower stability to GdnHCl and LDS^4^	higher kinetic stability^28^	same stability to SDS^22^
Size	similar (2–6 nm)^1^	similar (6.2 ± 0.5 nm)^4^	similar (4–5 nm)^3,12,28^	larger (unknown nm)^22^
Ability to destabilize cell membranes	higher^1,20,38,40,42^	higher^4,38,43^	higher^3,12^	higher^22^
Ability to cause cell dysfunction	higher^1,20,38−40,45^	higher^4,38,43^	higher^3,12^	higher^22^

**Table 2:** Experimental Evidence for Oligomer Toxicity (Toxic Relative to Nontoxic Oligomers; n.d. = Not Determined)

Technique	HypF-N	Aβ_42_	αSyn	Sup35 NM
MTT reduction	lower^1,45^	lower^4,43,45^	lower^3,12^	n.d
Ca^2+^ influx	higher^1,20,38−40,45^	higher^38,43,45^	higher^12,32^	n.d.
Calcein release	higher^20^	higher^45^	higher^3,12^	n.d.
Adenylate kinase release	n.d.	n.d.	n.d.	higher^22^
LDH release	higher^20^	higher^4^	n.d.	n.d.
ROS production	higher^20^	n.d.	higher^3,12^	n.d.
Lipoperoxidation	higher^20^	n.d.	n.d.	n.d.
Caspase-3 activation	higher^20^	n.d.	higher^12^	n.d.
Hoechst staining	higher^1^	n.d.	n.d.	n.d.
Propidium iodide staining	n.d.	n.d.	n.d.	higher^22^
Brightfield microscopy (visualized cell death)	n.d.	n.d.	n.d.	higher^22^
Choline acetyltransferase immunoreactivity in rat hippocampal slices	lower^20^	n.d.	n.d.	n.d.
Long-term potentiation in rat hippocampal slices	impaired^36^	n.d.	n.d.	n.d.
Spatial learning in rats (Morris Water Maze)	impaired^36^	n.d.	n.d.	n.d.
*C. elegans* motor phenotypes	impaired^37^	n.d.	impaired^37^	n.d.

Toxic oligomers can be targeted with therapeutics, as demonstrated by the antibody lecanemab in AD.^18^ A promising class of small molecules is aminosterols, which prevent the binding of toxic oligomers of HypF-N, Aβ_40_, Aβ_42_, and αSyn to cells, therein eliminating their toxicity.^51,52^ A variety of molecular chaperones shield hydrophobic regions on oligomers, therein attenuating their toxicity,^53^ and a wide variety of endogenous and exogenous inhibitors can reduce the number of toxic oligomeric aggregates formed through varied molecular mechanisms.^43,54^ In addition, oligomers have been implicated in other protein misfolding diseases, such as Huntington’s disease, spongiform encephalopathies, and type II diabetes. The continued study of existing oligomer pairs, as well as the development of new oligomer pairs for other proteins, will continue to facilitate a broader understanding of diverse protein misfolding diseases and identify nuances for each discrete pathology.
